# Mapping Second Chromosome Mutations to Defined Genomic Regions in *Drosophila melanogaster*

**DOI:** 10.1534/g3.117.300289

**Published:** 2017-10-24

**Authors:** Lily Kahsai, Kevin R. Cook

**Affiliations:** Bloomington *Drosophila* Stock Center, Department of Biology, Indiana University, Bloomington, Indiana 47405

**Keywords:** *Drosophila melanogaster*, mutation mapping, complementation, gene identification, forward genetics, mutant screen report

## Abstract

Hundreds of *Drosophila melanogaster* stocks are currently maintained at the Bloomington *Drosophila* Stock Center with mutations that have not been associated with sequence-defined genes. They have been preserved because they have interesting loss-of-function phenotypes. The experimental value of these mutations would be increased by tying them to specific genomic intervals so that geneticists can more easily associate them with annotated genes. Here, we report the mapping of 85 second chromosome complementation groups in the Bloomington collection to specific, small clusters of contiguous genes or individual genes in the sequenced genome. This information should prove valuable to *Drosophila* geneticists interested in processes associated with particular phenotypes and those searching for mutations affecting specific sequence-defined genes.

The phenotypes associated with mutations often provide insights into the functions of genes. Indeed, much of genetics research involves explaining how mutations give rise to phenotypes. Newer methods for inducing mutations such as transposon excision, homologous recombination, and CRISPR-based disruption are particularly good for deleting coding sequences. Such knockout mutations are undeniably important in understanding the cellular roles of genes, but other kinds of mutations—such as those that reduce gene expression or protein activity or affect only certain protein isoforms or domains—can be informative in ways that knockout mutations are not (Venken and Bellen 2014). While point mutations can now be engineered, they usually reflect the biases of the investigator. Older methods that induce mutations randomly by chemical or irradiation treatments have the advantage of probing gene function blindly. They can reveal novel protein structure–function relations and elicit unexpected phenotypes.

Knowing the importance of mutations, the Bloomington *Drosophila* Stock Center devotes considerable effort to maintaining stocks carrying mutations. Many of these mutations have been characterized phenotypically, but they have not yet been associated with sequence-defined genes. These stocks are potentially valuable, but they are requested infrequently. Geneticists interested in particular sequence-defined genes often do not consider them as a source of potential alleles, because the mutations are generally not tightly mapped. Likewise, geneticists interested in particular processes might be more likely to study mutations with relevant phenotypes if they knew they would be relatively easy to associate with sequence-defined genes. The usefulness and popularity of these stocks would be improved tremendously by anchoring these mutations to the genome sequence map so that their relationships to annotated genes could be recognized more readily.

Our ability to map mutations to specific genomic intervals in *Drosophila* improved enormously when simple techniques became available for generating chromosomal deletions with breakpoints known at single-nucleotide resolution. Three large-scale projects, including one conducted at the Bloomington *Drosophila* Stock Center, generated deletions with molecularly defined breakpoints (Parks *et al.* 2004; Ryder *et al.* 2007; Cook *et al.* 2012). Altogether, these deletions provide >98% genomic coverage and subdivide the genome into intervals of a median of nine genes (Cook *et al.* 2012). Using these deletions, mutations can now be mapped to very small chromosomal regions, or even single genes, with simple complementation tests. Mutations can often be mapped even more closely with follow-up complementation tests involving chromosomal duplications (Cook *et al.* 2010; Venken *et al.* 2010), or mutations affecting single genes.

We report here the localization of 77 complementation groups in the Bloomington *Drosophila* Stock Center collection to defined genomic intervals and the mapping of eight complementation groups to individual genes. This work ties these mutations to single genes or small groups of closely linked genes, and increases the value of an underutilized set of stocks.

## Materials and Methods

The data in this report came from fly crosses made on standard medium, reared under routine conditions, and evaluated by customary standards (details provided upon request). Genomic coordinates are given in terms of the Release 6 assembly, and gene annotations are those shown in the June 20, 2017 FlyBase release (FB2017_3). Supplemental Material, Table S1 in File S1 provides a list of stocks used and our sources.

### Data availability

The accompanying tables contain complete mapping data. Stocks may be obtained from the Bloomington *Drosophila* Stock Center or *Drosophila* Genomics and Genetics Resources at the Kyoto Institute of Technology as indicated in Table S1 in File S1.

## Results and Discussion

We identified a large set of second chromosome mutations in the Bloomington *Drosophila* Stock Center collection that had not been associated with annotated genes and used mapping information archived in FlyBase (http://flybase.org/), or recorded in publications to estimate the chromosomal positions of the mutations (Table S2 in File S1). We then made complementation crosses between stocks carrying the mutations and molecularly defined chromosomal deletions to place the mutations in defined genomic intervals that refine previous mapping (Table S3 in File S1). Subsequent crosses tested the mutations for allelism with mutations in sequence-defined genes. Table 1 summarizes our results. The number of candidate genes in each interval was initially determined by the overlap of deletions with transcribed gene regions. (We recognize this criterion is potentially misleading as it is possible for a deletion to remove gene regulatory regions and disrupt gene function even if transcribed gene regions are not deleted. Nevertheless, it is a reasonable and commonly employed practice for deletion studies.) From this total, we subtracted the number of genes with complementing mutations. (This criterion could also be misleading, because partial loss-of-function alleles can show intragenic complementation. Nevertheless, it is also a reasonable simplification for a preliminary mapping study.) We have provided a list of candidate sequence-defined genes for each complementation group in Table S4 in File S1. Table S5 in File S1 provides a full list of the informative mapping crosses. In every cross, we had experimental evidence indicating that both stocks were valid as follows. Every mutation being mapped failed to complement at least one deletion. Most stocks used to map the mutations were validated with independent control crosses to stocks carrying relevant, previously characterized, loss-of-function mutations or chromosomal deletions (Table S6 in File S1). For a dozen deletion stocks, noncomplementation of the deletion with one of the mutations we were mapping was taken as evidence the stock was intact, and no independent control cross was undertaken.

**Table 1:** Mapping complementation groups to specific genomic intervals

Complementation group	Genomic interval from deletion mapping	Complementing mutations	Noncomplementing mutations	Candidate genes*^a^*	Comments
*abb*	2R:23666959;23713811			13	
*bhe*		*Gs1^1^*, *Sam-S^1^*	*dbr^EP9^*, *l*(*2*)*gl^4^*		Our complementation tests with deletions (Table S4 in File S1) and these mutations indicate *bhe^1^* is a multigene, terminal deletion (*Df*(*2L*)*bhe*) with a breakpoint between *dbr* and *Sam-S*. Polytene analysis showed a breakpoint at 21A5-B1. This is consistent with J. Kennison’s observation of at least one *bhe* allele failing to complement *l*(*2*)*gl* (cited in Lindsley and Zimm 1992). The mutant embryonic phenotype likely results from the disruption of several genes.
*blo*	2R:8733630;8898753	*RyR^Q3878X^*, *sns^MI12892^*, *sns^MI03001^*		11	
*bub*	2L:7702880;7718010			6	
*c*	2R:15918423;15950652			10	Our data are consistent with unpublished identifications of *c* as *Strn-Mlck* by Rodriguez (2004) and E. Spana and E. Green (personal communication).
*cass*	2L:17473293;17482011		*Aac11^k06710^*	1	These results show *cass* is the same gene as *Aac11*.
*dw-24E*	2L:4361214;4403405	*Tps1^k08903^*		9	Szidonya and Reuter (1988) mapped *dw-24E* left of *Tps1*, reducing candidates to five. Curry (1939) showed *l*(*2*)*cg^1^* was originally present in the nearby *dpy* to *cl* region, but our crosses to spanning deletions showed no lethality, suggesting it was removed before the current stock was established.
*eay*	2R:18173570;18230554			11	
*flz*	2R:8976399;9031045	*Np^MI00240^*, *Np^MI10279^*	*CG8213^MI04680^*	1	Our identification of *flz* as *CG8213* is consistent with the independent, unpublished results of Anne Uv (cited in Geberemedhin 2011).
*Frd*	2R:23001651;23068684	*twi^1^*		6	Our mapping is based on the recessive lethality of *Frd^1^*. *Frd^1^* mutants carry an intragenic deletion in *PPO3* (Sugumaran and Chase 2004).
*fs*(*2*)*abc*	2R:15375176;15386324	*dup^PA77^*		1	
*fs*(*2*)*lto3*	2L:19464056;19517610			14	
*fs*(*2*)*ltoQE45*	2L:3656901;3713827			24	
*fs*(*2*)*ltoRM7*	2R:23385467;23395914			2	
*hum*	2R:7395885;7447410	*so^D^*		18	Heitzler *et al.* (1993) mapped *hum* left of *so*, reducing candidates to nine.
*l*(*2*)*21Ba*	2L:67365;159063	*Sam-S^R23^*, *Sam-S^1^*, *Gs1^1^*		23	Caggese *et al.* (1988) showed *l*(*2*)*21Ba* is not the same gene as *Gs1*. Larsson *et al.* (1996) mapped *l*(*2)21Ba* right of *Sam-S* and left of *Gs1*, reducing candidates to five.
*l*(*2*)*23Ab*	2L:2677694;2753125			9	Littleton and Bellen (1994) mapped *l*(*2*)*23Ab* left of *Pgk*, reducing candidates to seven.
*l*(*2*)*24Dc*	2L:4162968;4197800			5	
*l*(*2*)*24Dd*	2L:4031318;4162968	*ed^1^*		5	Szidonya and Reuter (1988) showed *l*(*2*)*24Dc* is not the same gene as *ed*.
*l*(*2*)*24De*	2L:4162968;4197800			5	
*l*(*2*)*25Cg*	2L:5073453;5145500			5	Szidonya and Reuter (1988) showed *l*(*2*)*25Cg* is not the same gene as *Msp300*, reducing candidates to four.
*l*(*2*)*34Db*	2L:13721648;13800829			16	John Roote reported *l*(*2*)*34Db* maps right of *P{lacW}TM9SF4^k07245^* (http://flybase.org/reports/FBrf0129261.html) and left of *Df*(*2L*)*Exel7059* (personal communication), reducing candidates to nine. This is consistent with the tentative conclusion that *l*(*2*)*34Db* is the same gene as *Sec71* (Ashburner *et al.* 1999).
*l*(*2*)*35De*	2L:15821840;15912343			15	Our data maps *l*(*2*)*35De* to one of two intervals, but this interval is consistent with the mapping of Ashburner *et al.* (1999).
*l*(*2*)*36Ba*	2L:16824908;16886557			14	
*l*(*2*)*36Fe*	2L:18606977;18617225			3	
*l*(*2*)*37Ab*	2L:18673286;18689053	*msl-1^kmB^*		4	
*l*(*2*)*37Ac*	2L:18753432..18753444;18795820			2	
*l*(*2*)*37Dc*	2L:19438065;19452918			4	
*l*(*2*)*37De*	2L:19438065;19452918			4	
*l*(*2*)*37Di*	2L:19464056;19517610	*swm^37Dh-1^*		13	Brittnacher and Ganetzky (1983) showed *l*(*2*)*37Di* is not the same gene as *swm*.
*l*(*2*)*37Ea*	2L:19517610;19528383			5	
*l*(*2*)*37Fb*	2L:19576108..19576133;19586375			6	Rutledge *et al.* (1992) and Gay and Contamine (1993) showed *l*(*2*)*37Fb* is not the same gene as *spi*, reducing candidates to five.
*l*(*2*)*37Fc*	2L:19576108..19576133;19586375			6	Rutledge *et al.* (1992) and Gay and Contamine (1993) showed *l*(*2*)*37Fc* is not the same gene as *spi*, reducing candidates to five.
*l*(*2*)*37Fe*	2L:19586375;19753324	*Lar^13.2^*		7	Butler *et al.* (2001) showed *l*(*2*)*37Fe* is not the same gene as *scw*, reducing candidates to six.
*l*(*2*)*38Ab*	2L:20085397;20120504	*neb^k05702^*		7	
*l*(*2*)*38Db*	2L:20449190..20458307;20638580			24–26	
*l*(*2*)*38Eb*	2L:20680624;20770538	*Hr38^02306^*, *Fs*(*2*)*Ket^RX3^*		11	Butler *et al.* (2001) and Kozlova *et al.* (2009) showed *l*(*2*)*38Eb* is not the same gene as *dia*, reducing candidates to 10.
*l*(*2*)*38EFb*	2L:20831386;20851900			6	
*l*(*2*)*43Ba*	2R:7187225;7326951			9	Heitzler *et al.* (1993) showed *l*(*2*)*43Ba* is not the same gene as *pwn*, reducing candidates to eight.
*l*(*2*)*43Cc*	2R:7493197;7533553	*dpa^EY04015^*, *dpa^1^*		11	Heitzler *et al.* (1993) mapped *l*(*2*)*43Cc* right of *dpa*, reducing candidates to nine. MacIver *et al.* (1998) tentatively identified *l*(*2*)*43Cc* as *didum*.
*l*(*2*)*43Da*	2R:7493197;7533553	*dpa^1^*, *dpa^EY04015^*		12	Heitzler *et al.* (1993) mapped *l*(*2*)*43Da* right of *dpa*, reducing candidates to 10.
*l*(*2*)*43Db*	2R:7493197;7533553	*dpa^EY04015^*		12	Heitzler *et al.* (1993) mapped *l*(*2*)*43Db* right of *dpa*, reducing candidates to 10.
*l*(*2*)*43Ef*	2R:7665795;7708707	*tor^4^*, *U2A^1^*		9	Heitzler *et al.* (1993) showed *l*(*2*)*43Ef* is not the same gene as *U2A* or *tor* and maps right of *U2A*. Nagengast and Salz (2001) showed a *U2A* transgene did not rescue *l*(*2*)*43Ef* mutations. This reduces candidates to four.
*l*(*2*)*43Eg*	2R:7665795;7708707	*U2A^1^*		10	Heitzler *et al.* (1993) showed *l*(*2*)*43Eg* maps right of *U2A* and is not the same gene as *tor*, reducing candidates to four.
*l*(*2*)*46Ca*	2R:9875312;9922003..9927457	*tea^1755^*	*Etf-QO^f05640^*	1	These results show *l*(*2*)*46Ca* is the same gene as *Etf-QO*.
*l*(*2*)*46Cb*	2R:9875312;9922003..9927457	*Etf-QO^f05640^*, *tea^1755^*		8	O’Brien *et al.* (1994) showed *l*(*2*)*46Cb* is not the same gene as *FMRFa*, reducing candidates to seven.
*l*(*2*)*46Cd*	2R:9958120;10025288	*eve^3^*, *eve^5^*, *Pal1^ta-1^*, *eIF3j^k13906^*		9	
*l*(*2*)*46Db*	2R:9959818;10025288	*eve^3^*, *eve^5^*, *Pal1^ta-1^*, *eIF3j^k13906^*, *TER94^k15502^*, *TER94^EY03486^*	*TER94^03775^*, *TER94^26-8^*, *TER94^22-30^*	1	These results show *l*(*2*)*46Db* is the same gene as *TER94*, even though *l*(*2*)*46Db^26^* shows a complex complementation pattern with other *TER94* alleles.
*l*(*2*)*46Dc*	2R:10025288;10030539			3	
*l*(*2*)*46Dd*	2R:10030539;10078293			16	
*l*(*2*)*46De*	2R:9959818;10025288..10025310	*eve^5^*, *eIF3j^k13906^*		8	O’Brien *et al.* (1994) mapped *l*(*2*)*43De* right of *eve*, reducing candidates to four.
*l*(*2*)*46Df*	2R:10030539;10078293			16	
*l*(*2*)*49Dc*	2R:12894105..12894116;12940453..12949055			12–13	
*l*(*2*)*49Fa*	2R:13197974..13198492;13219347..13219349			10	Lasko and Pardue (1988) showed *l*(*2*)*49Fa* is not the same gene as *Orc3*, reducing candidates to nine.
*l*(*2*)*49Fg*	2R:13219130;13249241			14	Lasko and Pardue (1988) mapped *l*(*2*)*49Fg* left of *Dp*, reducing candidates to three.
*l*(*2*)*51Ea*	2R:15218008;15262942		*scb^2^*	1	These results show *l*(*2*)*51Ea* is the same gene as *scb*.
*l*(*2*)*57Ba*	2R:21000163;21056798			8	
*l*(*2*)*57Bd*	2R:21056798;21088247			12	
*l*(*2*)*57Cb*	2R:21056798;21088247			12	
*l*(*2*)*57Cc*	2R:21143577;21177310			12	J. M. O’Donnell *et al.* (1989) suggested *l*(*2*)*57Cc* is adjacent to *Pu* or overlaps it. Reynaud *et al.* (1999) suggested *l*(*2*)*57Cc* is not the same gene as *Xpd*.
*l*(*2*)*57Cd*	2R:21143577;21177310			12	J. M. O’Donnell *et al.* (1989) suggested *l*(*2*)*57Cd* is adjacent to *Pu* or overlaps it. Reynaud *et al.* (1999) suggested *l*(*2*)*57Cd* is not the same gene as *Xpd*.
*l*(*2*)*57Ce*	2R:21180990;21215223			9	J. O’Donnell *et al.* (1989) showed *l*(*2*)*57Ce* is not the same as *tud*. This reduces candidates to eight.
*l*(*2*)*57Db*	2R:21301798;21341647			23	
*l*(*2*)*57Eb*	2R:21497209;21607081			16	J. O’Donnell *et al.* (1989), Price *et al.* (1989), and Schejter and Shilo (1989) showed *l*(*2*)*57Eb* is not the same gene as *Egfr*, reducing candidates to 15.
*l*(*2*)*57Ec*	2R:21497209;21607081			16	J. O’Donnell *et al.* (1989) showed *l*(*2*)*57Ec* is not the same gene as *Egfr*, reducing candidates to 15.
*l*(*2*)*DA2*	2L:9522946;9560489			7	
*l*(*2*)*DB2*	2L:9897536;9908459			2	
*l*(*2*)*DB4*	2L:9205076;9388129			20	Lane and Kalderon (1993) showed *l*(*2*)*DB4* is not the same gene as *Cks30A*, reducing candidates to 19.
*l*(*2*)*FE3*			*hoip^k07104^*	1	These results show *l*(*2*)*FE3* is the same gene as *hoip*. It was mapped with cytologically defined deletions (Table S4 in File S1), but not molecularly defined deletions, so no genomic interval is given.
*l*(*2*)*N7-6*	2L:9622987;9699225			5	
*l*(*2*)*N7-8*	2L:9205076;9388129	*Cks30A^RA74^*		19	Lane and Kalderon (1993) showed *l*(*2*)*N7-8* is not the same gene as *Cks30A*.
*l*(*2*)*PC4-A*	2R:18051197;18118348	*stau^1^*		11	
*l*(*2*)*PC4-D*	2R:17782032;17792649			4	Mohr and Gelbart (2002) mapped *l*(*2*)*PC4-D* to *Ubc10*, *CG5033* or *Dhit*, reducing candidates to two.
*l*(*2*)*PC4-M*	2R:17716263..17716471;17739901..17739916			11	
*l*(*2*)*PC4-P*	2R:18621522;18639268			6	
*l*(*2*)*PC4-Q*	2R:18621522;18639268			6	
*mat*(*2*)*syn-E*	2L:10349604;10381214			11	Our data place *mat*(*2*)*syn-E* in the same general region as Clegg *et al.* (1993), but they placed it right of *da* and *RpS27A*.
*moa*	2R:22729367;22764935			3	
*ms*(*2*)*35Eb*	2L:15912343;16025369			16	
*nrd*	2L:2517598..2551864;2621016			16–19	Littleton and Bellen (1994) mapped *nrd* right of *Drp1*, reducing candidates to five. Our results are compatible with *nrd^D20^* being associated with a small deletion as proposed by Littleton and Bellen.
*P{f^+^13}30B*	2L:9205076;9388129			20	These crosses show a lethal mutation (hereafter *l*(*2*)*30ABa^1^*) is caused by the *P{f^+^13}30B* insertion or is closely linked to it.
*pd*	2R:23811400;23844351	*shu^2^*		7	
*qui*	2R:23659139;23713811			12	
*sat*	2R:7493197;7533553		*Orc1^KO^*	1	These results show *sat* is the same gene as *Orc1*.
*sie*	2R:13034847;13159579			10	

a Excludes genes with complementing mutations from the set of contiguous genes defined by deletion breakpoints (Table S3 in File S1). Ranges reflect deletion breakpoint uncertainty. Candidate genes are listed in Table S4 in File S1.

We were able to map 77 complementation groups to the smallest chromosomal intervals possible using existing molecularly defined deletions (Table S3 in File S1). With follow-up complementation tests using existing point mutations and transposon insertions in annotated genes, we were able to map eight complementation groups to single annotated genes, but we did not exhaust all possible tests of this sort. In the final tally, we were able to map 84 of the 85 complementation groups to 26 genes or fewer. (We found the remaining complementation group, *bhe*, to be a multigene deletion.)

Table 2 summarizes information on the mutations mapped to single annotated genes, and shows the diversity of interesting genes affected. This work has identified the first nontransposon alleles of two genes (*Aac11* and *CG8213*), and has added potentially important EMS- or irradiation-induced alleles to the other genes. While we have not attempted to assess the allelic strength of most of the mutations, we know the female-sterile mutation *sat^SC46^* mapped to *Orc1* must be a partial loss-of-function allele because knockout alleles are recessive lethal (Park and Asano 2008). Mutations affecting a particular motif in the *Orc1* protein have been shown to cause the same defective eggshell phenotypes as *sat^SC46^* (Park and Asano 2012), which suggests it too is domain specific. This result illustrates the importance of point mutations maintained in stock for their loss-of-function phenotypes: they can reveal aspects of gene function that would not be apparent from the phenotypes of gene knockouts.

**Table 2:** Complementation groups mapped to single genes

Complementation group	Summary
*cassowary* (*cass*)	*cass* mutations were isolated as recessive lethal mutations that result in lack of adhesion between wing surfaces in homozygous mitotic clones (Prout *et al.* 1997). *cass* is allelic to *Aac11*, which encodes an inhibitor of apoptosis homologous to human *Apoptosis Inhibitor 5* (*API5*) (Morris *et al.* 2006).
*filzig* (*flz*)	*flz* mutations were isolated as recessive lethal mutations affecting the patterning of the embryonic cuticle (Nüsslein-Volhard *et al.* 1984). We found *flz* to be allelic to *CG8213*, which encodes a serine protease (Ross *et al.* 2003). Subsequently, we learned *flz* was also identified as *CG8213* by Anne Uv (unpublished results cited in Geberemedhin 2011).
*fs*(*2*)*abc*	*fs*(*2*)*abc* (*abnormal chromatin*) mutations were isolated as recessive maternal-effect lethals causing abnormal embryonic nuclear divisions and defective chorions (Schüpbach and Wieschaus 1989; Vessey *et al.* 1991). *fs*(*2*)*abc* is allelic to *SRPK*, which encodes a Serine–Arginine Protein Kinase necessary for dorsoventral egg patterning, karyosome formation, and meiotic divisions (Barbosa *et al.* 2007; Loh *et al.* 2012).
*l*(*2*)*46Ca*	The recessive lethal *l*(*2*)*46Ca* is allelic to *Electron transfer flavoprotein-ubiquinone oxidoreductase* (*Etf-QO*), which encodes a component of the electron-transport chain that generates ATP from the breakdown of fatty acids (Watmough and Frerman 2010).
*l*(*2*)*46Db*	The recessive lethal *l*(*2*)*46Db* is allelic to *TER94*, which encodes a chaperone that targets ubiquitin-tagged proteins to the proteasome (Meyer *et al.* 2012).
*l*(*2*)*FE3*	The recessive lethal *l*(*2*)*FE3* is allelic to *hoi-polloi* (*hoip*), which encodes a small nuclear ribonucleoprotein component of spliceosomes (Mount and Salz 2000).
*satin* (*sat*)	Schüpbach and Wieschaus (1991) showed homozygous *sat^SC46^* females lay eggs with thin eggshells. *sat* is allelic to *Origin recognition complex 1* (*Orc1*), which is needed for chorion gene amplification (Park and Asano 2008).
*l*(*2*)*51Ea*	The recessive lethal *l*(*2*)*51Ea* is allelic to *scab* (*scb*), which encodes an α integrin involved in cell adhesion (Stark *et al.* 1997).

In conclusion, we have refined the mapping of a large number of second chromosome mutations that have been preserved at the Bloomington *Drosophila* Stock Center for their mutant phenotypes. This information will provide *Drosophila* workers opportunities to make connections between these mutations and genes they might be studying in defined chromosomal regions.

## Supplementary Material

Supplemental material is available online at www.g3journal.org/lookup/suppl/doi:10.1534/g3.117.300289/-/DC1.
